# Ecology of the African Maize Stalk Borer, *Busseola fusca* (Lepidoptera: Noctuidae) with Special Reference to Insect-Plant Interactions

**DOI:** 10.3390/insects5030539

**Published:** 2014-07-08

**Authors:** Paul-André Calatayud, Bruno P. Le Ru, Johnnie van den Berg, Fritz Schulthess

**Affiliations:** 1 Institute de Recherche pour le Développement (IRD), UR 072, c/o *ICIPE* (African Insect Science for Food and Health), Nairobi 30772, Kenya; E-Mail: bleru@icipe.org; 2 CNRS UPR9034, Laboratoire Evolution, Génomes et Spéciation/Université Paris-Sud 11, Orsay Cedex 91405, France; 3 Unit for Environmental Sciences and Management, North-West University, Potchefstroom, 2520, South Africa; E-Mail: johnnie.vandenberg@nwu.ac.za; 4 Postfach 508, Chur 7000, Switzerland; E-Mail: Fritz.Schulthess@googlemail.com

**Keywords:** insect-plant interactions, insect distribution, Poaceae, wild grasses, oviposition, host preference, feeding status, plant damages, pest management

## Abstract

*Busseola fusca* (Lepidoptera: Noctuidae) is an important pest of maize and sorghum in sub-Saharan Africa. One century after its first description by Fuller in 1901, inaccurate information based on earlier reports are still propagated on its distribution (e.g., absent from the lower altitudes in East Africa) and host plant range (e.g., feeding on a large range of wild grass species). This review provides updated information on the biology, distribution and genetics of *B. fusca* with emphasis on insect-plant interactions. Related to this, new avenues of stem borer management are proposed.

## 1. Introduction

*Busseola fusca* was first mentioned as *Sesamia fusca* in a report by Fuller in 1901 [1] and described under the same name by Hampson in 1902 [2]. In 1953 African species of *Sesamia* and related genera were morpho-taxonomically revised and finally *S. fusca* was placed in the *Busseola* Thurau genus [3]. The first description of the oviposition site, eggs, larval behavior and damage symptoms caused by *B. fusca* stemmed from South Africa [4]. Since 1920, *B. fusca* is considered as an important pest of maize and sorghum in sub-Saharan Africa, and first recommendations on how to control this pest were given by Mally [5]. Since then, a plethora of information on its distribution, pest status and injuriousness were produced [6]. *B. fusca* is considered to be the most destructive lepidopteran pests of maize [6] and sorghum [7] in Africa. Estimates of crop losses vary greatly in different regions and agro-ecological zones. In Kenya alone, losses due to *B. fusca* damage on maize fluctuate around 14% on average [8], while in the humid forest zone of Cameroon losses of around 40% are common in monocropped maize fields [9,10,11]. Currently, this pest still presents a major constraint to the production of maize in areas where they are abundant.

Inaccurate information from various reports is still propagated on its distribution [6] and host plant range [12,13]. Contrary to these reports, *B. fusca* does occur in the lower altitudes in East Africa and it feeds on only a few host plant species.

During the last decade, the interactions of this insect pest with plants (e.g., [14,15]) as well as its reproductive biology (e.g., [16,17,18,19] and genetics e.g., [20]) have been well documented. This review provides updated information on the biology, distribution, genetics, host plant range and preference as well as management of *B. fusca*. It largely considers studies conducted during the last two decades in Central, East and Southern Africa. In West Africa, *B. fusca* is only of economic importance in the dry agroecological zones and on sorghum only [21,22], and little information exists about the ecology and management of this pest in this region.

## 2. Biology and Reproduction

A good knowledge of the biology of *B. fusca* is a prerequisite for understanding how this species interacts with plants. Most of the information produced for *B. fusca* during the last century, which forms the basis of the knowledge of the biology and ecology of this pest, stemmed from South Africa However, since the majority of the studies in South Africa addressed *B. fusca* at high altitudes and in commercial farming systems, some aspects regarding its biology and interactions with the environment may differ from those in other agroecological zones. Furthermore, most of the following information on *B. fusca* biology and reproduction was obtained on maize plants.

### 2.1. Eggs

*Busseola fusca* females oviposit a highly variable number (from 100 up to 800) of round and flattened eggs in batches [18,23,24]. The batches are laid behind the vertical edges of leaf sheaths of pre-tasseling plants and also, but rarely, underneath the outer husk leaves of ears [5]. Van Rensburg and colleagues [24] recorded eggs on 12- to 16-week old plants, but only when these were planted very late in the season. It appears that the position at which the eggs are found correlates with the developmental stage of the plant, and with increasing plant age, egg batches are increasingly found higher up on the plant [24]. Van Rensburg and colleagues [24] noted that leaf sheaths fitted more loosely around stems as plants gets older, and that females preferred the sheaths of youngest unfolded leaves for oviposition.

Although it is rare to find more than one *B. fusca* egg batch per plant, van Rensburg and colleagues [24] reported cases of between 2 and 4 egg batches per plant. They however attributed this to extremely high population pressure at late planting dates.

In South Africa, with its unimodal rainfall pattern allowing for one crop per annum, it was also observed that egg batches of spring moth generation are smaller than those of summer moths [18,25]. A possible explanation is that body reserves of spring moths are smaller than those of the summer moths since the former would have utilized reserves during diapause. Similarly, Usua [26] in Nigeria reported that spring moths laid approximately 65% fewer eggs than summer moths. Field studies during which more than a thousand egg batches were collected in South Africa, showed that the average size of an egg batch of 1st and 2nd generation females were 22 and 33 eggs respectively [25]. Results from van Rensburg and colleagues [25] indicate that a single moth lays 7–8 egg batches, an observation supported by Ingram [27], and Kruger and colleagues [18] under laboratory conditions. *Busseola fusca* fecundity has not been studied in areas with bimodal rainfall distribution, which allows for more than one cropping season per annum.

### 2.2. Larvae

Larvae hatch after about one week and they migrate first to the whorl where they feed on young and tender leaves deep inside the whorl. In contrast to stem borer species from the *Sesamia* and *Chilo* genera, young *B. fusca* larvae do not consume any leaf tissue outside of the whorls of plants. Larvae can remain in the whorls of especially older plants (6–8 weeks old) up to the 4th instar [28]. From the 3rd instar onwards, larvae migrate to the lower parts of the plant where they penetrate into the stem. Some larvae do however migrate away from natal plants with approximately 4% of larvae leaving the natal plant immediately after hatching [28]. The larval stage lasts between 31 and 50 days [18,29,30] and consists of 7–8 instars with a minimum of 6 [23,31]. More recently, continuous observations of larvae on an artificial diet indicated that, under optimum environmental conditions (25 °C and 50%–60% r.h.), the larval stage consisted of 5 stages and was completed during approximately 35 days. Additional instars were observed when the conditions were suboptimal or when larvae went into diapause [32].

Although it is well known that *B. fusca* undergoes a facultative diapause consisting mostly of a larval quiescence [24,33,34], several issues around this survival mechanism remain unclear. Although Okuda [35] showed that water contact is a significant factor terminating diapause, the mechanisms explaining diapause physiology in *B. fusca* have not been fully elucidated.

### 2.3. Adults

#### 2.3.1. Emergence and Life Duration

The mean sex ratio of *B. fusca* is 1:1.1 (male:female) [18,19,36]. The adults emerge about 13–14 days after pupation [18,29,30] and they emerge mostly between sunset and midnight [29,37]. Most males emerge before onset of the scotophase, while most females do so one hour later [30,37]. The average life span of moths ranges between 8 and 10 days [16,18,34].

#### 2.3.2. Pheromones

Only the females emit pheromones. Males and females exhibit simple and rapid courtship behaviour without any particular characteristic events [38]. The sex pheromone of *B. fusca* females was first identified as a mixture of (Z)-11-tetradecen-1-yl acetate (Z11–14: Ac), (E)-11-tetradecen-1-yl acetate (E11–14: Ac), and (Z)-9-tetradecen-1-yl acetate (Z9-14: Ac) [39]. More recently, an additional pheromone component, (Z)-11-hexadecen-1-yl acetate was identified and when added to the aforementioned three-component synthetic blend resulted in improved attraction of males [40].

#### 2.3.3. Mating

The females start calling a few hours after emergence, indicating absence of a sexual maturation time [37]. The calling behaviour generally commences during the fourth hour after the onset of the scotophase but it is slightly delayed for females having emerged the same night as compared to older females [37]. Mating starts within a few hours after moth emergence [23]. Moreover, mating occurs generally during the first six hours of the night, and the males can mate several times but only once per night [36]. A single spermatophore is generally sufficient to fertilize all eggs of a female throughout her life span [36], indicating that polyandry is not obligatory and not necessary. Laboratory studies also showed that female calling behaviour and male attraction was not influenced by the presence of plants, irrespective if it was a host or non-host [41].

**Figure 1:**
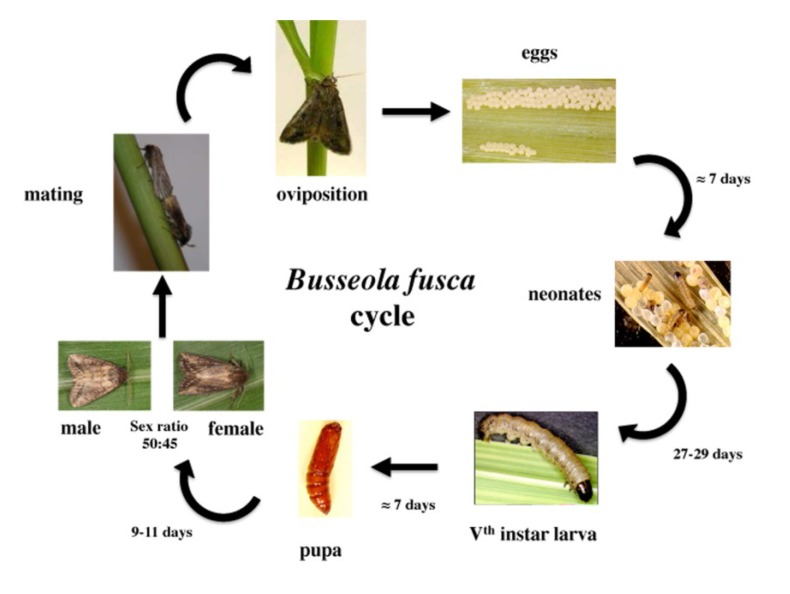
Biological cycle of *Busseola fusca* under optimal environmental conditions on artificial diet (photos on mating and oviposition from [42]).

The oviposition period lasts for 3–4 nights [23]. It commences during the first night after mating, peaks during the second and then gradually decreases until the fifth night [37]. A summary of the life cycle of *B. fusca* with updated information is provided in Figure 1.

## 3. Damage Symptoms

*Busseola fusca* larvae damage all plant parts of the cultivated crops they attack. In South Africa, before the advent of genetically modified (GM) *Bt*-maize, *B. fusca* often occurred in mixed populations with another stem borer, *Chilo partellus* (Lepidoptera: Crambidae) [7]. *Busseola fusca*, *S*. *calamistis* and *C*. *partellus* larvae were often observed in mixed populations in the same planting as well as in individual plants. Multiple species attacks are also frequently observed in the different agro-climatic areas in Kenya [43]. Mixed infestations of *B. fusca* and *C. partellus* were also observed in the Highveld region of South Africa [44,45]. Although Kfir [46] speculated that *B. fusca* tended to avoid plants, which were previously infested by *C. partellus*, both species are often recorded on the same plant [7,43,44]. In the humid forest zone of Cameroon, mixed populations of *B. fusca*, *S. calamistis* and the pyralid *Eldana saccharina* Walker are common [47].

## 4. Geographical Distribution

*Busseola fusca* occurs throughout sub-Saharan Africa [6] but not in Zanzibar and Madagascar [48]. In East Africa, from 1905 [4] until recently, *B. fusca* was thought to occur only at high altitudes. In West Africa, it is of minor importance in the southern humid zones, but it is the dominant species on cultivated sorghum in the northern dry savannah zones [49]. In Central Africa, field surveys indicated that *B. fusca* was the dominant species of all agroecological zones from sea level to the highlands, and from the humid forest to the dry savannah zones [10]. Similarly, in East Africa *B. fusca* occurs in all agroecological zones from the lowland semi arid and arid savannahs to the highland African wet mountain forests, though it is only predominant above 1500 m a.s.l. [43,48,50,51,52]. A similar trend was also observed in South Africa where *B. fusca* occurs in inland low-laying areas [53], coastal areas [28,54] as well as in the mountain regions of Lesotho at an altitude of 2131 m a.s.l. [55].

## 5. Genetics

The genetic variation in *B. fusca* populations was assessed for the first time in 1997 [56]. A study using isozyme variation revealed a significant geographic differentiation between populations collected at Lake Victoria and Trans Nzoia in Kenya. However, no genetic differentiation in relation to the host plant on which *B. fusca* larvae were collected from could be found, suggesting free movements of *B. fusca* populations among different host plants within their ecosystems. Nine years later, genetic analyses and nested clade phylogeographic analyses (NCPA) separated African *B. fusca* populations into three mitochondrial clades, one from West Africa (W), and two [Kenya I (KI) and Kenya II (KII)] from East and Central Africa [20,57]. The same studies also concluded that there was no discernible effects on the geographic patterns observed in the *B. fusca* mitochondrial genome in relation to the host plant where the larvae were collected from. It was also demonstrated that clade KII had a greater geographic distribution than KI; it was found in Eritrea, Ethiopia, Kenya, Uganda, Cameroon, Tanzania, Zambia, Malawi, Mozambique, Zimbabwe and South Africa, whereas KI is restricted to Uganda, Kenya, Ethiopia and Eritrea [20,57]. A recent study carried out on the genetic structure and origin of *B. fusca* populations in Cameroon, based on the comparison of the cytochrome b sequences, showed a moderate but significant structuring between the Guineo-Congolian rain forest and Afromontane vegetation mosaics populations. Molecular diversity in the Guineo-Congolian rain forest zone was lower than that in Afromontane vegetation, which indicates that the former was most likely the starting point for the colonization of other zones in Cameroon [58].

The phenomenon of genetic differences between *B. fusca* populations could also shed light on the association between *B. fusca* and one of its most common natural enemies, *Cotesia sesamiae* (Hymenoptera: Braconidae). It could, in theory, be possible that the greater expansion of clade KII as compared to KI was due to differences in their reproductive potential and/or in their resistance to *C. sesamiae*, which is the most common larval parasitoid of *B. fusca* in East and Central Africa [17] as well as South Africa [59]. It was shown that despite their long time co-existence, for example in Kenya, KII and KI conserve biological differences in terms of time of calling, fecundity, fertility and resistance against the larval parasitoid *C. sesamiae*, which explains the wider distribution of KII as compared to KI in that country [17]. However, the pheromone blends of KI and KII cannot be differentiated [40]. The same study showed that the sex pheromone mixture and the male responses did not play a role in either reproductive isolation between the sympatric clades, nor for the divergence of the specific mate recognition systems (SMRS) between the allopatric east and western populations.

For a better understanding of *B. fusca* phylogeography, microsatellite loci were for the first time isolated and characterized in this species in 2005 [60]. This allowed for phylogeographic studies, for example to assess the effect of contiguous migration at different climatic periods on genetic differentiation in *B. fusca* [61].

## 6. Host Plant Range

From the *Handbook of Information* on *B. fusca* published in 1992 [49], *B. fusca* was considered as a species feeding not only on maize, cultivated and wild sorghum but also on many wild grasses like *P. purpureum*, *Panicum maximum*, *Hyparrhenia rufa*, *Rottboellia exaltata* and *Phragmites* sp. This list of wild host plants was extended later to other genera of grasses such as *Cymbopogon*, *Cynodon*, *Echinochloa*, *Setaria*, *Sporobolus* and *Tripsacum*, accounting for a total of 18 species of wild grasses [12,13,56,62]. *Busseola fusca* was never reported on dicotyledonous plants or other graminaceous crop plants (e.g., rice, wheat or sugarcane) that are cultivated in the areas of Africa where this pest occurs. More recently, improved taxonomical expertise using morphological and molecular tools allowed final conclusions about the low diversity of wild hosts for this stemborer, showing that the host plant range of *B. fusca* was not as wide as reported previously [48,50,63,64,65,66,67,68,69]. This discrepancy is probably due to previous misidentifications, as several species of *Busseola*, notably *B. phaia* Bowden, *B. segeta* Bowden and *Busseola nairobica* Le Ru [70], which closely resemble *B. fusca*, are frequently found on wild host plants around cereal crops (Table 1). *Busseola segeta* also occurs in maize in Western Kenya [71] and *B. phaia* is commonly found in maize in highland areas in the south western highlands (Iringa, and Njombe regions) of Tanzania [72,73]. In fact *B. fusca* accounts for only 13.6% of all the *Busseola* larvae collected on wild hosts in surveys conducted over the past decade. Although the above mentioned *Busseola* species are found only on grasses, the host plant range of *B. fusca* is less diversified with only 7 host plant species, compared to 16 and 19 for *B. segeta* and *B. phaia*, respectively. The following host plant species can therefore be removed from the initial and older host plant list of *B. fusca*: *Cynodon dactylon*, *Hyparrhenia cymbaria*, *Hyparrhenia filipendula*, *Hyparrhenia rufa*, *Panicum deustum*, *Pennisetum massaicum*, *Pennisetum polystachion*, *Phragmites karka*, *Rottboellia cochinchinensis*, *Setaria incrassata*, and *Sporobolus pyramidalis.*

**Table 1:** A summary of *Busseola* spp. and their host plant identified during surveys in 15 countries, on 197 plant species belonging to Poeaceae, Cyperaceae, Typhaceae, Juncaceae and Thurniaceae families. Surveys were conducted between 2003–2013 in sub-Saharan Africa, following the sampling procedure described by Le Ru and colleagues [63] (total number of noctuid stem borer larvae collected was 48,910) [73].

Host plants	Number of *Busseola fusca*	Number of *Busseola nairobica*	Number of *Busseola phaia*	Number of *Busseola segeta*	Countries	Total number of individuals collected
*Andropogon gayanus* Kunth	0	0	16	0	TZ	16
*Arundo donax* L.	500	0	0	0	ER, ET, SA	500
*Cymbopogon giganteus* Chiov.	8	0	0	0	ZB	8
*Cymbopogon nardus* (L.) Rendle	66	0	37	17	ET, KE, SA, TZ	120
*Cynodon dactylon* (L.) Pers.	0	0	0	7	KE	7
*Echinochloa pyramidalis* (Lam.) Hitchc. & Chase	0	0	4	74	KE, UG	78
*Euchlaena mexicana* Schrader	0	0	25	2	KE, UG	27
*Heteropogon contortus* (L.) P. Beauv. ex Roem. & Schult]	0	0	1	0	TZ	1
*Hyparrhenia diplandra* (Hack.) Stapf	0	0	3	0	TZ	3
*Hyparrhenia dregeana* (Nees) Stapf ex Stent	0	0	1	0	TZ	1
*Hyparrhenia papillides* (A. Rich.) Stapf	0	0	0	5	KE, UG	5
*Hyparrhenia schimperi* (Hochst. ex A. Rich.)	0	0	7	0	MO	7
*Hyparrhenia* sp.	0	0	30	0	TZ	30
*Melinis minutiflora* P. Beauv.	0	0	0	2	CA	2
*Panicum atrosanguineum* Hochst. ex A. Rich.	0	0	45	0	TZ	45
*Panicum deustum* Thunb.	0	2,559	0	199	ET, KE, UG	2,758
*Panicum mapalende* Pilg.	0	0	0	10	JE, TZ	10
*Panicum maximum* Jacq.	47	0	72	221	ET, KE, MO, RC, DRC, RW, UG	340
*Pennisetum clandestinum* Chiov.	0	0	0	38	CA, ET, KE	38
*Pennisetum macrourum* Trin.	0	0	7	18	KE, TZ	25
*Pennisetum purpureum* Shumach.	59	25	507	1,184	CA, ER, ET, KE, MO, UG, DRC, RW, TZ, ZB	1,775
*Pennisetum unisetum* (Nees) Benth	0	0	1	17	KE, ZB	18
*Phragmites mauritianus* Kunth.	0	0	11	0	TZ	11
*Setaria megaphylla* (Steud.) T. Duran & Schinz	3	0	10	2	ET, RC, TZ	15
*Setaria plicatillis* (Hochst.) Engl.	0	3	0	0	KE	3
*Setaria sphacelata* (Schumach.) Moss	0	0	3	12	CA, TZ	15
*Snowdenia polystachya* (Fresen.) Pilg.	0	0	2	0	TZ	2
*Sorghum arundinaceum* (Desv.) Stapf	135	0	0	10	KE, SA, TZ, UG	145
*Sporobolus macranthelus* Chiov.	0	0	2	0	TZ	2
Total number of individuals	818	2,587	784	1,818		6,007

CA: Cameroon; ER: Eritrea; ET: Ethiopia; KE: Kenya; MO: Mozambique; UG: Uganda; RC: R Congo; DRC: Democratic Republic of Congo; RW: Rwanda; SA: South Africa; TZ: Tanzania; ZB: Zambia.

In addition to maize and sorghum species [63,64,65,66,67,68], *B. fusca* can also be found on *Arundo donax* in Ethiopia, Eritrea and South Africa, and sporadically on *P. maximum*, *P. purpureum*, *Cymbopogon nardus*, *C. giganteus* and *Setaria megaphylla* in most countries (Table 1). Under laboratory and semi-field conditions however, *B. fusca* has been reported to lay large numbers of eggs on several varieties of *P. purpureum*, with the number of eggs laid per variety as well as larval survival being negatively correlated with the number of trichomes present on leaves of these varieties [69]. *Busseola fusca* has also been reported to infest *P. purpureum* under field conditions in South Africa [74]. In Kenya, *B. fusca* is rarely found to infest *A. donax* and *P. purpureum* compared to Ethiopia and South Africa, respectively. This can be due to locally adapted *B. fusca* populations, which are able to infest those plant species in those countries, though there still exists the possibility that the species have not been properly identified. For example, in Cameroon, follow-up studies and identification of borers by Ndemah *et al*. [64] showed that the reported frequent occurrence of *B. fusca* on *P. purpureum* in earlier studies [62,74] was the result of misidentification of the species.

## 7. Host Plant Preference and Selection

The host plant preference and selection of oviposition site are determined by adults (involving the females) while larvae are responsible for colonization and feeding. As in many other lepidopteran species [75,76], the success of *B. fusca* to recognize/select and colonize its host is based on the interaction between its sensory systems and the physical/chemical characteristics of its immediate environment.

### 7.1. Adults Behaviour and Preferences

#### 7.1.1. Moth Flight Patterns

Light and pheromone traps have been used extensively to study the flight patterns of *B. fusca* in South Africa [77]. Generally, in areas where only one rainy season occurs, distinct flight patterns are observed. Moth numbers in pheromone and light traps show less discernible patterns in areas where maize is cultivated throughout the year [28].

Based on the work of Du Plessis and Lea [78] in South Africa, it was known in the early 1970s that more than one generation of moths occurred in a season and that early infestations in a given season were derived from late infestations of the preceding season. Infestation patterns also vary between localities and are associated with the rainfall and temperature gradient existing from east to west in the greater production area in South Africa. Among the most noteworthy findings in South Africa were that there were three seasonal moth flights, which varied geographically in both magnitude and duration from east to west [79]; that moths gave oviposition preference to 4-week old plants [24,80]; and that moth numbers vary in accordance with the rainfall pattern [25]. The first flight commences during early spring after the first good rains. The first and second flights are separated by a distinct period in December during which no moths occur. In an attempt to assist producers in identifying potentially hazardous on-farm flight levels, a pheromone trapping system was developed during the 1980s [81]. The system was, however, shown to be unreliable during periods of pronounced moth activity due to poor competition of the synthetic pheromone with the natural product [82].

#### 7.1.2. Behavioural Bases of Host Plant Selection

The different behavioural steps leading to host selection and oviposition have been well described in *B. fusca* [15]. Similar to other noctuids, the behavioural steps leading to oviposition by a gravid moth follow a sequential pattern involving searching, orientation, encounter, landing, surface evaluation, and acceptance. Before landing, plant volatiles influence the female orientation, indicating from a distance the suitability of the plant species [83]; the female antennae bear numerous multiporous trichoidea sensilla able to collect volatiles [84]. Thereafter, the visual cues are also involved in the female’s orientation and landing [15]. It is after landing that the final decision for oviposition takes place. Thereby, the female typically sweeps her ovipositor on the plant surface as if evaluating the suitability of the plant, simultaneously touching it with the tips of her antennae, and then, if the plant is accepted, oviposition takes place. The tip of each antenna bears several uniporous sensilla able to taste the plant’s surface [84]. In addition, the ovipositor bears about nine uniporous chemosensory sensilla (*i.e.*, taste receptors) located within the inner border of the ventral surface of each lobe of this organ [84]. Combinations of tactile and gustatory stimuli from the plant are received during the “ovipositor sweep” behaviour. During this behavioural step, the claws at the distal part of the ovipositor leave small injuries on the plant surface, which are deep enough to liberate inner plant cuticular compounds, which differ between plant species (e.g., between host and non-host plants) [85]. These compounds are perceived by the taste receptors on the ovipositor, which then activate the appropriate behaviour (acceptance or rejection) depending on the nature/composition of these cuticular chemicals.

Like all noctuid borer species, *B. fusca* females oviposit egg batches between the leaf sheath and the stem [16]. *Busseola fusca* prefers to oviposit inside leaf sheaths of the youngest fully unfolded leaves [24]. In choice tests, *B. fusca* moths show preference for maize to sorghum plants of similar sizes [69]. It can therefore be concluded that the physical properties of the leaf sheath and stem play also a crucial role in plant acceptance for oviposition. In fact, *B. fusca* prefers to oviposit on waxy plant species [86] and do not oviposit at all on *Melinis minutiflora*, a species with glandular trichomes [87]. It was also shown that 3–6 weeks old maize plants are most attractive for oviposition [24,79]. Thus, *B. fusca* prefers pre-tasseling plants; oviposition rarely occurs on older maize plants [24], but if so, the insect lays batches on the upper part of the plants where the leaf sheaths are young and soft [88]. Oviposition on maize plants in the post-anthesis period has been reported by van Rensburg and colleagues [25] but, when provided with a choice, moths prefer plants during the vegetative stages of development.

A significant correlation of r = 0.69 was obtained between stalk circumference and egg number in maize, confirming in part the previously found preference of *B. fusca* moths for thick stemmed plants [14,59,80].

All these reports and observations suggest that plant physical cues, such as surface texture (e.g., pubescence), plant size (e.g., stem diameter), and leaf sheath rigidity, strongly influence the acceptability by *B. fusca* of a host species or plant part. More recently, it has been shown that *B. fusca* do not prefer to oviposit on plants with very small stem diameter and they prefer to oviposit on plants with non-pubescent or smooth surfaces over pubescent or rough surfaces [14]. Pubescence and rough surfaces significantly affect the behavioural steps leading to oviposition since it interferes with the ovipositor sweep process necessary to find a suitable oviposition site. In addition, the rigidity of the support that the leaf sheath provides also influences the proper insertion of the ovipositor for egg deposition. It can be concluded that oviposition acceptance in *B. fusca* is very likely caused by evolved mechanisms of oviposition site selection, that is, suitable oviposition sites are restricted to the gaps between the leaf sheath and the stem, and, hence, rigidity and pubescence of the stem or leaf sheath will affect oviposition [14]. Oviposition patterns, host selection and to a lesser extent larval distribution on plants seem to be closely related to crop phenology. In field studies on grain sorghum in South Africa, oviposition on both main stems and tillers reached a maximum at six to eight weeks after plant emergence [89]. This differs from the known pattern in maize of three to five weeks, and could be ascribed to the difference in growth rates of the two crops. Elongation of grain sorghum stems is slower, while stalks of maize are generally thicker and thus favour oviposition at earlier crop growth stages. The period of oviposition is extended in grain sorghum, possibly due to tillering, which provided leaf sheaths of suitable tightness over a longer period of crop development than in maize [89].

### 7.2. Larval Behaviour and Preferences

#### 7.2.1. Larval Migration Patterns

*Busseola fusca* larvae migrate throughout all larval stages [24]. This migration commences immediately after egg hatch and ceases during the last instar when larvae prepare pupal cells in which they become pupae, or go into diapause.

Clear patterns in the intra-seasonal progression of larval infestations have been described by van Rensburg and colleagues [25]. Although a small proportion of larvae migrate off plants immediately after hatching, the great majority (81%) of larvae up to the 4th instar remain in the whorl [25,90]. The low degree of damage caused by stem borer larvae to whorl leaves of wild hosts indicates that they do not feed in whorls for extended periods of time [91]. In late-infested maize and sorghum, 1st instar larvae may commence feeding on silk of ears, panicles or in young emerging panicles for some time before migrating and commencing feeding inside ears or stems. Van Rensburg and colleagues [25] reported that on late infested maize there was no clear preference for 1st instars to feed on ears instead of stems, and that occurrence of 1st instars on ears is most likely a function of time of plant growth stage at the time of oviposition and of larvae searching for soft tissue and shelter.

Migration does not cease after the larvae leave plant whorls to feed inside maize stems. Larvae migrate until the 6th instar, a behaviour that is clearly density dependent. Migration of late-instar larvae between plants also increases the likelihood of parasitism and predation. In South Africa, large numbers of 5th–6th instar larvae of *B. fusca*, parasitized by *C. sesamiae*, are often observed inside the last whorl leaves of plants, when maize plants commence anthesis and flowers emerge [92]. In areas such as the Highveld region of southern Africa, where *B. fusca* goes into facultative diapause for a period of at least 5 months, only one 6th instar larvae occurs per stem base, a few cm below soil level [25]. In warmer areas such as Zimbabwe, Smithers [93] reported that *B. fusca* goes into diapause in the lower part of the stem, 25–60 cm above soil surface.

Van Rensburg and colleagues [59] reported that up to 70% of larvae may migrate to adjacent rows over a 5-week period, and that the incidence of plants remaining with a single larva at this time may be as high as 67% [25]. This extent of occurrences of a single larva per stem in spite of the pseudo-aggregated oviposition behaviour illustrates the high migration potential of *B. fusca.* It also explains the patchy infestation pattern of *B. fusca* in the field [5,21,78,93] and the increased percentage of plants damaged by larvae over time.

#### 7.2.2. Behavioural Bases of Host Plant Selection

Host selection in phytophagous insects is generally determined by the adults. However, in many Lepidoptera species the larvae can engage in host plant selection [94]. After hatching underneath the leaf sheath, *B. fusca* neonate larvae ascend to the whorl, where they either feed on the leaves or disperse via “ballooning-off” [16]. This dispersal phenomenon is generally density dependent and might be influenced by host plant quality. After feeding in the leaf whorl, 3rd to 4th instar larvae descend and bore into the plant stem. Older larvae may also migrate in search of other or more suitable host plants [16,24]. Generally, lepidopteran larvae display food preferences *via* a phenomenon driven by chemoreceptors located on the mouthparts [95]. Like other Lepidoptera species, *B. fusca* larvae possess sensory structures able to detect plant compounds, including volatiles [96]. Although the antennae of the larvae are short and simple, they bear three multiporous cone-shaped basiconic sensilla able to detect volatiles. In fact, the 3rd instar larvae are able to recognize the odours of their host plant by a distance [97]. The larvae possess also on their maxillary galeae two uniporous styloconic sensilla, which are contact chemoreceptors. They have also maxillary palps having eight small basiconic sensilla at the tip, which were also found to be gustatory [96]. Plant sugars are often considered as primary feeding stimuli, involved among the compounds that induce the host plant acceptance by herbivorous insects [76,77]. It was recently shown that sucrose is a feeding stimulant and positively influences food choice by *B. fusca* larvae, whereas turanose (an isomer of sucrose), as a phagodeterrent, negatively contributes to larval food choice [98]. The uniporous styloconic sensilla of the maxillary galeae are able to detect both sugars but the lateral styloconic appears more sensitive to sucrose at low concentrations whereas the medial styloconic is more sensitive to turanose. These findings indicate that the balance in concentrations of these sugars somehow influences the overall host plant choice made by the larvae.

Among the most important factors determining larval choice of host plant might be differences in silicon (Si) content. In higher plants, silicon levels range between 0.1%–10% on a dry weight basis and they are generally higher in grasses than in dicotyledonous plants [99]. Plant resistance to insects, pathogens or abiotic factors has been shown to be related to the level of accumulation and polymerization of Si in tissues [100]. For *B. fusca*, it has been observed that Si in plant epidermal cells appears to provide a physical barrier by increasing leaf abrasion, which subsequently increases wearing off of the mandiblesof *B. fusca* larvae, which physically deter larval feeding [101]. Consequently, *B. fusca* larvae prefer to feed on grasses that have low levels of Si.

## 8. Integrated Management of *Busseola fusca*

Since *B. fusca* is an important pest of maize in sub-Saharan Africa, a wide range of methods have been researched, tested and implemented to manage this pest. These include among others control by pesticides, cultural practices, host plant resistance as well as biological control agents [6]. Since the focus of this review is on insect-plant interactions, an extensive review of *B. fusca* management is not provided here. Management aspects that relate to insect-plant interactions are however addressed below.

Cultural control is a long-established method of modifying the habitat to make the environment unfavourable for the survival and reproduction of pests. Moreover, it is the most relevant and economic method of stem borer control available for resource-poor farmers in Africa [102]. This management strategy, considered the first line of defence against pests and among the oldest traditional practices, includes techniques such as destruction of crop residues, intercropping, crop rotation, manipulation of planting dates, tillage methods and improvement of soil fertility. In addition, the aim of these control techniques is to reduce rather than eradicate pest populations and it can be used in conjunction with other methods. Diagnostic work in West Africa indicated that increased plant diversity in (mixed cropping) and around (wild habitats) maize fields, or improvement of soil fertility via integration of grain legumes or cover crops as short fallow, or provision of nitrogen fertilizer or silicon (Si) could influence *B. fusca* infestation levels [10,103]. In addition to cultural control, host plant resistance, genetically modified *Bt* maize and chemical control offer potential options for pest management.

### 8.1. Mixed Cropping

Field trials in West Africa showed that, depending on the crop association (*i.e.*, maize with cassava, cowpea or soybean) and planting pattern, mixed cropping reduced *B. fusca* egg densities by 41.2%–67.0%, and larval densities by 44.4%–83.0%. As a consequence, maize yield losses due to stem borer damage were 1.8–3.0 times higher in monocrops than intercrops [9,104,105].

According to Aiyer [106] in Finch and Collier [107] the lower number of specialised herbivores (such as *B. fusca*) on the main crop in mixed cropping systems might be as a consequence of the non-host companion crop (i) reducing host finding by the ovipositing moth (disruptive crop hypothesis) [9,106,107,108], (ii) acting as alternative host plants (trap crop hypothesis) [86], or (iii) serving as a repellent to the pest [88,108]. In addition, Vandermeer [108] lists the natural enemy hypothesis in which the intercrop attracts more parasitoids and predators. For *B. fusca* there is no field evidence (*i.e.*, oviposition on non-hosts as proposed by Khan and colleagues [87] supporting the trap crop hypothesis. Furthermore, female B. fusca moths inspect plants with their antennae and ovipositor before ovipositing [15], and it is, thus, very unlikely that they would oviposit on a host that does not guarantee survival of their offspring. Furthermore, Finch and Collier [107] state that repellent chemicals appear to be effective over only a few centimetres. The repellent crop would therefore not prevent the insect from entering a field, but once in the field the insect would be diverted onto the target crop and so nullify the crop-protection benefit of the companion crop. Concerning the natural enemy hypothesis, in field trials in Cameroon and Benin, mixed cropping systems yielded considerably higher parasitism of *B. fusca* eggs than monocrops [104,105] but it was shown that this was due to a negative relationship between host densities and parasitism common for insect parasitoids. Thus, the disruptive crop hypothesis, which includes several mechanisms (physical obstruction, visual camouflage, masking of host plant odours, appropriate/inappropriate landing) is most likely the most suitable one explaining the lower *B. fusca* infestations on maize mixed with non-host companion crops. Finch and Collier [107] also suggest that non-host companion crops disrupt host-plant finding by providing insects with a choice of host (appropriate) and non-host (inappropriate) plants on which to land, and their research has shown that [107,109], for intercropping to be effective, insects must land on the non-host plants as well, thereby wasting time. On the other hand, mixed cropping with non-host companion crops appears not to be effective against borers such as the pyralid *Eldana saccharina* Walker, which, in contrast to the noctuids *B. fusca* or *Sesamia calamistis* Hampson attack the plant after tasseling [104,110]. This suggests that companion crops produce a “masking” effect (*i.e.*, visual camouflage or masking of host odour) [107,109], which affects host finding of the moth, and that this effect disappears as the plant grows taller. Intercropping of maize and sorghum with non-host crops has also been shown to be effective in reducing borer numbers in South Africa [102].

### 8.2. The Role of Wild Habitats

Country-wide surveys in West Africa and Cameroon showed that stemborer densities within maize fields significantly decreased with abundance of wild grasses around the field [10,22,103]. It was suggested that those grasses act as trap plants or as habitats that perennate natural enemies during the off-season. In subsequent field trials, however, the planting of border rows with grass species abundant in the area had a weak or no effect on stemborer densities and per plant yield of maize, and per area yields were reduced because of the smaller area planted to maize [52,62,111].

Various authors [48,50,63,64,65,66,67,68,69] showed that the host plant range of *B. fusca* is narrow and with the exception of *Sorghum arundinaceum* (Desv.) Stapf, the noctuid was either not or rarely found on the plant species planted as border rows, thus their role as trap plants for *B. fusca* is questionable. This was put in evidence by Calatayud and colleagues [15] for Napier grass *Pennisetum purpureum* Schumach. Finch and Collier [107] suggested that for the crambid *Chilo partellus* (Swinhoe), *P. purpureum* acts mostly as a physical obstruction rather than being a trap plant as proposed by Khan and colleagues [87]. In the trials by Ndemah and colleagues [51,62] and Matama-Kauma and colleagues [111], there were some effects during the second and third season after planting the border rows but never during the first season corroborating the physical obstruction hypothesis by Finch and Collier [107,109]. It was concluded that the grass density was not sufficient to have a reliable effect and planting more border rows was not an affordable technology.

In Africa, uncontrolled burning of fields and adjacent wild habitats during the dry season to clear land from bush-fallow in preparation for the first crop probably has a considerable impact on especially natural enemies. In some treatments of the border row experiments, the planting of grasses enhanced parasitism by the braconid larval parasitoid *C. sesamiae* and the scelionid egg parasitoids, *Telenomus* spp. In Cameroon, *Telenomus* spp. cause high mortality of *B. fusca* eggs and a positive relationship was found between maize yields and egg parasitism [103]. As *B. fusca* diapauses during the off-season, wild habitats, which harbour alternative hosts of parasitoids, are important in perennating natural enemies during times when the main pest is scarce [112,113,114]. Recent surveys in South Africa showed that although natural habitats provided refuges for some parasitoid species of stem borers, stem borer parasitism was generally low in wild host plants [115]. Hence, it appears important to protect wild habitats surrounding maize fields.

### 8.3. The Effect of Soil Fertility on Pest Infestation and Yields

Host plant feeding status, which is affected by factors such as fertilizer and water stress, affects stem borer infestation levels, damage levels and subsequent yield losses.

Fertilizer trials in Cameroon showed that stemborer abundance at the early growth stage increased with increasing N level [47]. By contrast, grain yield losses decreased and they were 11 to 18 times higher with 0 kgN/ha compared to 120 kgN/ha. Similar results were obtained earlier by Sétamou and colleagues [116] and Mgoo and colleagues [117] for *S. calamistis* and *C. partellus*, respectively. In addition, Chabi-Olaye and colleagues [11] compared a continuous maize production system with crop sequence systems, in which maize followed a grain legume (cowpea, soybean), a cover crop (mucuna, pigeon pea) or a bush fallow. As in the fertilizer trial, maize in the rotation systems had 1.5–2 times higher borer densities at the early growth than continuous maize production systems. Whereby average yields increased in the crop sequences, grain yield losses were 2 to 3 times higher in the continuous maize production system than in the crop sequences of grain legumes with maize, and 4.5–11 times higher compared to maize after cover crops. In both the fertilizer and grain legume trials, larval “disappearance” rate at older stages of maize increased with increasing nutritional status (N mainly) of the plant. Whether this was due to a higher mortality or density dependent emigration could not be determined with the experimental setup. It is concluded that an increased nutritional status of the plants lead to an increase in borer attacks at the early stage of plant growth, but it also improved plant vigour, resulting finally in a net benefit for the plant and grain yield. Increasing soil N also increases larval parasitism probably by enhancing the nutritional quality of the plant and its insect host [118,119,120], indicating a multi trophic level interaction. Improving soil fertility can thus be a very effective means of an integrated control strategy for *B. fusca*. Similar results have been obtained with the *B. fusca* and *C. partellus* stemborer complex in South Africa [121]. It should however be born in mind that damage-yield loss relationships are complex and that, while stem borer infestation levels and damage may be influenced by nutritional status of plants, high-yielding plants may suffer proportionately more yield loss [121].

### 8.4. Host-Plant Resistance

Host-plant resistance has potential to provide effective control of *B. fusca* [122] and has been indicated to be compatible with other control methods [123]. However, maize varieties resistant to this pest are still not available in Africa [6].

Evaluation of maize and sorghum genotypes for resistance to *B. fusca* was performed in South Africa after the development of a method to collect large numbers of overwintering larvae [122]. Using this method, winter-collected *B. fusca* larvae can be kept in diapause in the laboratory for extended periods and the diapause can be terminated at will to provide moths and large numbers of neonate larvae for artificial infestation of plants in the field [122].

Maize inbred lines resistant to North American lepidopteran pests of maize were evaluated in South Africa and they have been shown to be highly resistant to *B. fusca* [124]. Viable resistance to *B. fusca* was later identified in several lines developed by CIMMYT in Mexico [125]. After mass screenings and elite line developments, 42 stem borer resistant maize breeding red lines were released in South Africa during 2004 [126]. All of this was, however, eclipsed by advances in molecular genetics and development of genetically modified maize.

The value that stem borer resistance in sorghum hybrids could have in suppression of pest populations was shown by van den Berg [127]. However, screening of more than a 1800 sorghum breeding lines for resistance to *B. fusca* showed that antibiosis resistance levels were low and that tolerance to damage and recovery resistance were the mechanisms that resulted in reduced yield losses in some lines [128,129,130].

### 8.5. Genetically Modified Maize

Genetically modified (GM) maize expressing insecticidal Cry proteins (*Bt*-maize) have been deployed with success against *B. fusca* in South Africa until 2006 when the 1st case of resistance was reported [131]. The reasons provided by farmers for the high adoption rate of *Bt*-maize were largely given as ease of management [132]. Nevertheless, *Bt*-resistant *B. fusca* populations have been reported throughout the maize production region of South Africa [132]. The resistance was shown not to be recessive as previously assumed [133]. GM maize will be approved for control of several lepidopteran stem borer species in Africa within the next few years. Due to the unique nature of African farming systems (e.g., seed sharing practices) this will provide new challenges to managing this pest in subsistence farming systems [134].

Knowledge on *B. fusca* moth and larval behavior, such as those summarized in this paper, are critical in development of insect resistance management (IRM) strategies against stem borers in Africa.

### 8.6. Plant-Derived Pesticides

Plant-derived pesticides are one of the alternatives to chemicals and are considered environmentally friendly. The efficacy of plant-derived pesticides is largely demonstrated not only in grain storage insects (see [135] for review) but also to control various insect species by the widely used extracts from the Indian neem tree, *Azadirachta indica* A. Juss. (Meliaceae) [136]. Several studies showing the potential to use plant-derived pesticides to control *B. fusca* were reported during the last two decades (e.g., [135,137,138,139]).

## 9. Conclusions

Extensive studies in Central, East and Southern Africa during the last 20 years showed that *B. fusca* occurs in all agroecological zones from the lowlands to the highlands and that the host plant range was much narrower than previously thought. This narrow host plant range was due to physical and chemical plant characteristics that influence the interactions between *B. fusca* and its host plant. These include:

(i) Physical characteristics: Stem circumference, plant pubescence and the tightness of the leaf sheath strongly influence host plant acceptance by gravid females for oviposition. High levels of Si in plant epidermal cells provide a physical barrier by increasing leaf abrasion, which subsequently increases wearing off of *B. fusca* larvae mandibles, which physically deter larval feeding.
(ii) Chemical characteristics: Plant volatiles are used by the gravid females for host plant finding. After landing, the cuticular chemical composition of the plant surface conditions the host plant acceptance by the ovipositing females. In addition, the balance of sucrose and turanose in leaves influences the host plant choice made by the larvae.

Current research aims to understand how stem borer pests colonize cultivated maize plants and how the colonization is affected by interactions with adults at both the intra and interspecific level to develop new methods to monitor pest populations and even to control them. It is well established that plant diversity in (mixed cropping) and around (wild habitats) a maize field, or improvement of soil fertility via integration of grain legumes or cover crops as short fallow, or provision of nitrogen fertilizer influence *B. fusca* infestations and injuriousness.

Some of the new avenues of stem borer management will consider also volatiles released by plants as selection criteria and by targeting certain volatiles by selection or by genetic engineering of the chemical signal released by the host plant [140]. In parallel, it is important to understand the interactions of maize with other crops and plants and with soil elements (e.g., N, Si) not only in terms of volatile emissions but of the overall plant chemistry, including non-volatile compounds and metabolites. All these studies imply a good knowledge on the chemical ecology of the different interactions (soil-plants; plants-plants and plants-insects).
